# Comparison of different rating scales for the use in Delphi studies: different scales lead to different consensus and show different test-retest reliability

**DOI:** 10.1186/s12874-020-0912-8

**Published:** 2020-02-10

**Authors:** Toni Lange, Christian Kopkow, Jörg Lützner, Klaus-Peter Günther, Sascha Gravius, Hanns-Peter Scharf, Johannes Stöve, Richard Wagner, Jochen Schmitt

**Affiliations:** 1 Center for Evidence-based Healthcare, University Hospital and Faculty of Medicine Carl Gustav Carus, TU Dresden, Germany; 2 University Center of Orthopaedics and Traumatology, University Medicine Carl Gustav Carus Dresden, TU Dresden, Germany; 3 Department of Therapy Science I, Brandenburg Technical University Cottbus, Senftenberg, Germany; 4 Orthopaedic and Trauma Surgery Centre (OUZ), Medical Faculty Mannheim, Heidelberg University, Mannheim, Germany; 5 Department of Orthopaedic and Trauma Surgery, St. Marien- und St. Annastiftskrankenhaus, Ludwigshafen, Germany; 6 Agaplesion Markus Krankenhaus, Frankfurt am Main, Germany

**Keywords:** Delphi, Consensus, Reliability, Outcomes, Rating scales, Treatment goals

## Abstract

**Background:**

Consensus-orientated Delphi studies are increasingly used in various areas of medical research using a variety of different rating scales and criteria for reaching consensus. We explored the influence of using three different rating scales and different consensus criteria on the results for reaching consensus and assessed the test-retest reliability of these scales within a study aimed at identification of global treatment goals for total knee arthroplasty (TKA).

**Methods:**

We conducted a two-stage study consisting of two surveys and consecutively included patients scheduled for TKA from five German hospitals. Patients were asked to rate 19 potential treatment goals on different rating scales (three-point, five-point, nine-point). Surveys were conducted within a 2 week period prior to TKA, order of questions (scales and treatment goals) was randomized.

**Results:**

Eighty patients (mean age 68 ± 10 years; 70% females) completed both surveys. Different rating scales (three-point, five-point and nine-point rating scale) lead to different consensus despite moderate to high correlation between rating scales (r = 0.65 to 0.74). Final consensus was highly influenced by the choice of rating scale with 14 (three-point), 6 (five-point), 15 (nine-point) out of 19 treatment goals reaching the pre-defined 75% consensus threshold. The number of goals reaching consensus also highly varied between rating scales for other consensus thresholds. Overall, concordance differed between the three-point (percent agreement [p] = 88.5%, weighted kappa [k] = 0.63), five-point (*p* = 75.3%, k = 0.47) and nine-point scale (*p* = 67.8%, k = 0.78).

**Conclusion:**

This study provides evidence that consensus depends on the rating scale and consensus threshold within one population. The test-retest reliability of the three rating scales investigated differs substantially between individual treatment goals. This variation in reliability can become a potential source of bias in consensus studies. In our setting aimed at capturing patients’ treatment goals for TKA, the three-point scale proves to be the most reasonable choice, as its translation into the clinical context is the most straightforward among the scales. Researchers conducting Delphi studies should be aware that final consensus is substantially influenced by the choice of rating scale and consensus criteria.

## Background

In a patient-centered, value-based health care system, medical decision making for elective surgery relies on the evaluation of the likelihood to achieve certain treatment goals. These goals are specified individually with respect to the patient’s needs. The likelihood to achieve these goals through surgery is estimated by the physician, resulting in the indication for elective surgery.

Up to 20% of patients are dissatisfied or not completely satisfied with the outcome of total knee arthroplasty (TKA) [1]. Therefore, the multi-perspective EKIT (Evidence and Consensus based Indication for Total Knee Arthroplasty) initiative [2] has been established to identify indication criteria for the German healthcare system in order to minimize the amount of unsatisfying treatments of knee osteoarthritis (OA) via TKA. According to the EKIT initiative, a consensus-based set on global treatment goals was essential to identify factors that determine and can modify the likelihood to achieve patients’ treatment goals. These factors form the external evidence for the consensus process of the indication criteria. The consensus on the set of global treatment goals was determined using the Delphi technique according to the a priori defined methodological framework of EKIT [3].

The Delphi technique has been developed by the RAND Corporation [4]. This technique is an iterative multistage consensus process in which individual opinions are combined into a group consensus [5, 6]. Several rounds of surveys (typically two or three) are conducted in Delphi studies, including anonymous feedback and possibility to adjust ratings with the goal of reaching a consensus [4–8]. Delphi consensus procedures have become widely used in various disciplines of medical research [9, 10], and are commonly used in the development of clinical practice guidelines and quality indicators [6], but also in the development of reporting guidelines [11], criteria for the appropriateness of interventions [8, 12] or core outcome sets (COS) [13, 14]. Despite the wide use, reporting standards and preregistered analysis plans for Delphi studies are currently lacking [15].

Vastly differing approaches are used to define final consensus [9], including the use of different aggregation methods and different rating scales. Previous methodological research on Delphi studies focused on the consensus definition (e.g. “consensus is reached in case of” > 80% equal ratings/ 90% of ratings scoring 7+ on a *nine-point scale*) [9], panel composition [16], question orders [17] and feedback strategies [18, 19]. To our knowledge, the use of different scales (e.g. the *nine-point scale*, a yes/no scale) regarding the impact on consensus has not been evaluated broadly.

While the nine-point scale is frequently used in Delphi studies [8, 9, 20, 21], the five-points scale is established in the field of expectation surveys [22–25]. In an interdisciplinary context, it is thus not a priori clear, which scale to choose for the purpose of the study. As a consequence, different scales could be chosen by different researchers which leads to the question how reliable the findings are and to which extent they depend on the chosen scale. The reliability of rating scales, however, forms the basis for any content validity. Accessing this reliability is a crucial step towards the optimal mapping of patient opinions.

In the context of identifying global treatment goals for TKA, we chose to compare three different rating scales. These are the *nine-point scale*, which is widely used for consensus processes [9, 13], the *five-point scale*, which has already been used in the area of patient expectation surveys [26], and a context-based *three-point scale*. In order to develop a set of global treatment goals, we investigated the impact of these three rating scales on final consensus as an embedded study within the framework of the EKIT initiative.

The objective of this study was (1) to explore the influence of rating scales and different consensus criteria on the selection of treatment goal(s) and (2) to investigate the test-retest reliability of the rating of these treatment goal(s) on different scales used in Delphi studies.

## Methods

### Patients and recruiting procedure

Target population were patients with knee OA scheduled for TKA. In order to ensure representativeness of the target population, eligible patients were recruited consecutively within routine care in five orthopaedic hospitals across Germany.

Eligible patients were invited to participate in this study during their orthopaedic consultation and were informed that there would be two surveys. Patients who withdrew their consent or submitted incomplete survey record sets (complete-case-analysis) were excluded.

### Study procedure

The study consisted of two surveys. In the first survey, a questionnaire and a pre-paid self-addressed envelope were handed out to each patient during the orthopaedic consultation. Patients willing to participate were asked to send the completed questionnaire including a signed letter of consent back to the study center by mail. The second survey was handed to each patient at inpatient admission before undergoing TKA, consisting of the second questionnaire and again a pre-paid self-addressed envelope. Patients completed the questionnaire prior to TKA and were asked to send it back to the study center. In this way, both surveys were answered before undergoing TKA. The re-test of the survey was conducted within 2 days to 2 weeks after the first survey. This period was chosen because (1) the lower limit (2 days) was based on different internal hospital procedures and (2) the upper limit (2 weeks) was chosen to minimize risk of bias through a change of patients’ opinions due to progression or acute events of OA.

Patients were asked to evaluate 19 pre-defined treatment goals using three different rating scales. Therefore, for each of the scales, a set of 19 questions formed a question block, with each question associated to a treatment goal. The order of question blocks was randomized between both surveys. The order of the 19 questions within each question block was randomized in both surveys, too. This was to minimize response behavior influenced by previous ratings (aiming to initiate a new test situation for each type of scale).

Data of each patient were fed into a database using Microsoft Access forms. In order to fulfil the requirements of data security and privacy protection, collection/storage of data and statistical analyses were conducted by different individuals. The study was conducted from beginning of February until the end of September 2015.

### Questionnaire design

The first survey questionnaire consisted of questions on demographic data (e.g. age, sex, current employment status). The first and the second questionnaire (both specifically developed for this study) contained the three question blocks (Additional file 1). The treatment goals were selected based on a systematic literature review on the use of measurement instruments and outcome domains in studies with OA patients undergoing TKA [27]. Each goal belonged to one of the 19 domains “pain”, “range of motion (ROM)”, “strength”, “stability”, “malalignment”, “physical function”, “walking distance”, “walking stairs”, “activity of daily life”, “employability”, “physical activity”, “sex life”, “quality of life”, “global health status”, “participation in social life”, “implant survival”, “no side effects”, “duration of hospitalization” and “preventing secondary impairments”. In the last question of the questionnaire, we asked which scale the respondent preferred.

### Rating scales

**Fig. 1:**
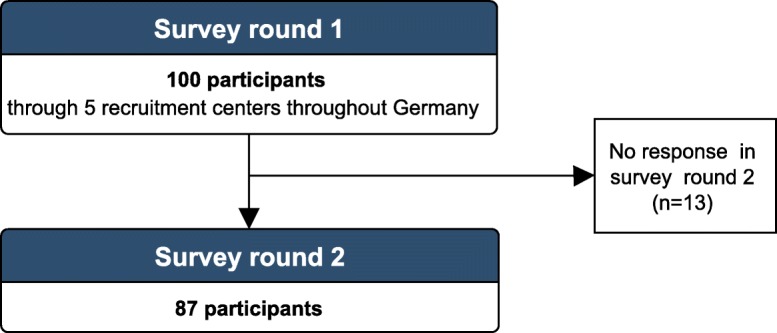
Flow chart

Three different rating scales were used simultaneously to measure patient expectations regarding outcome after TKA. These were the *three-point*, the *five-point* and the *nine-point scale* (Fig. 1). The answers to all 19 questions on treatment goals were recorded using these three scales, which were categorized as following.

The *three-point scale* includes the response categories: “main goal”, “secondary goal”, and “no goal”. The “*main goal*” was described to the patient as the outcome, which must be achieved through TKA intervention, otherwise the joint replacement is considered as unsuccessful. The “*secondary goal*” was described as desirable but not necessary for the success of the TKA. Finally, “*no goal*” was defined as an unimportant or inapplicable result. Our clinical experience was the reason for the choice of this scale.

The *five-point scale* was developed by Mancuso, et al. [26] and is frequently used in the field of orthopaedic surgery expectations [22–25]. The scale includes the following response categories: “very important”, “somewhat important”, “a little important”, “I do not expect this” and “this does not apply to me”.

The *nine-point scale* has been used within multiple Delphi procedures in biomedical research [8, 9, 20, 21]. It consists of a numeric range from 1 to 9. In addition, in this study, the range of 1 to 9 was divided in three sections and the questionnaires were labelled accordingly as categories 9, 8, 7 = “*important*”; 6, 5, 4 = “*important, but not critical*”; 3, 2, 1 = “*not important*”.

Via the *three-point scale,* we intended to detect patients’ “*main goals*”, whereas with using the *five-point* and the *nine-point scale,* we intended to classify the importance of treatment goals.

### Ethical considerations

This study was performed in accordance with the ethical standards of the Declaration of Helsinki [28] and with ethical approval by the Ethical Committee of the Faculty of Medicine of the TU Dresden in November 2014 (EK 423112014).

### Sample size calculation

We conducted an a priori sample size calculation as recommended for reliability studies [29–31] using the R package „kappaSize “with the function CI3Cats and the parameters kappa0 = 0.5, kappaL = 0.3, kappaU = 0.7, c (0.7,0.2,0.1), raters = 2, alpha = 0.05. This calculation resulted in a minimum sample size of 78 patients to evaluate test-retest reliability. Based on our clinical experience and the feasibility study conducted previously, we assumed that in the *three-point scale* the first category (“*main goal*”) has an occurrence probability of 70% and that the other categories (“*secondary goal*” and “*no goal”*) occur with a probability of 20 and 10%. The minimal expected kappa coefficient (*k*) should be detected with *k = 0.5* in an interval of ±0.2; the calculation of the sample size is therefore conservative. Since the sample size would decrease with an increasing number of categories, the result for the *three-point scale* sets a lower limit to the sample size [32]. Hence, a sample size calculation was not required for the other two scales as they have more categories in comparison to the three-point scale.

Note that in the process of analyzing the study the focus of the two main aims changed as it turned out that the appropriate choice of scale was far from trivial. Our aim (2) was initially termed aim (1). Hence, the sample size calculation was performed for this aim. Thus, the results of our (current) aim (1) are exploratory.

### Statistical analysis

All statistical analyses were performed using R Version 3.2.0 (The R Project for Statistical Computing, Vienna, Austria) and RStudio Version 0.99.491 (RStudio, Inc., Boston, MA).

Within this study, we investigated, for each scale, the proportion of treatment goals that reached consensus. In addition, we have calculated the correlation between the three rating scales for each survey. Between the two surveys, we calculated the reliability of the test-retest for each scale. Demographic data and the preferences for a given rating scale were analyzed using frequency distributions.

#### Correlation of rating scales

Correlation and similarity of rating behavior between the three ordinal ratings scales were calculated using *Spearman’s rho* in the first survey. Correlation coefficients range from − 1 to 1 (from maximum negative to maximum positive). According to Hinkle, et al. [33], correlation coefficients can be interpreted as following: 0.00 to 0.30: “*negligible correlation*”; 0.30 to 0.50: “*low correlation*”; 0.50 to 0.70 “*moderate correlation*”; 0.70 to 0.90: “*high correlation*”; and 0.90 to 1.00: “*very high correlation*”.

#### Effects of using different rating scales on consensus

##### (A) Effect of different ratings scales on the percentage main goals

The aim of the Delphi study [34] related to this manuscript was to identify global “*main treatment goals*” of patients, who were scheduled for TKA. To investigate the influence of different rating scales on the resulting consensus, we compared the proportion of proposed treatment goals that reached consensus on “*main goals*” between the three scales, as a prerequisite of the actual Delphi study [34]. To enable comparability of the results, treatment goals rated on the *five-point scale* as “*very important*” or rather on the *nine-point scale* as “9, 8, 7” were mapped as a “*main goal”*.

##### (B) Effect of different rating scales on percentage consensus using different thresholds

Different thresholds for consensus were tested to investigate the robustness of the result for overall consensus. These thresholds were defined that at least 60, 70, 75, 80% or 90% of patients had to rate the proposed treatment as a “*main goal*”.

#### Test-retest reliability of different rating scales

The test-retest reliability describes the capability of a measurement instrument to differentiate among subjects or objects under repeated assessment conditions that are similar [35]. The value of a single reliability measure is limited [36–38] and several statistical approaches for evaluation have been proposed [35]. In accordance with De Vet, et al. [39], we reported in this paper both, absolute (percentage agreement, number of changes in percent) and relative (weighted kappa coefficient, with quadratic weights) reliability measures.

According to the classification of Landis, et al. [40], kappa can be interpreted as the following: *k* values < 0.00 indicate poor, 0.00 to 0.20 slight, 0.21 to 0.40 fair, 0.41 to 0.60 moderate, 0.61 to 0.80 substantial and > 0.81 almost perfect agreement. However, the appropriate degree of agreement is context specific [35]. Therefore, proposed classification of Landis, et al. [40] should be considered as a rule of thumb and used with caution.

In order to allow for qualitative comparison and to adjust reliability measures, an equal number of rating categories is needed. Therefore, values of the *five-point* and *nine-point scale* were transformed into a *three-point scale*. The items “v*ery important*” (*five-point scale*) and “9, 8, 7” (*nine-point scale*) are transformed into a category called as “*main goal”*. Furthermore, “a *little important*”, “*somewhat important*” (*five-point scale*) and “5, 6, 7” (*nine-point scale*) are called “*secondary goal*”. “*I do not expect this*” and “*this does not apply to me*” from the *five-point scale* and “*3, 2, 1*” from the *nine-point scale* are called “*no goal*”. The transformed scales are named “five-point*” and “nine-point*” scale and we analyzed their reliability. As a sensitivity analysis, we computed the reliability measures for these scales in addition to the untransformed scales. Note that the absolute numbers depend on our choice of transformation.

## Results

### Patients

**Fig. 2:**
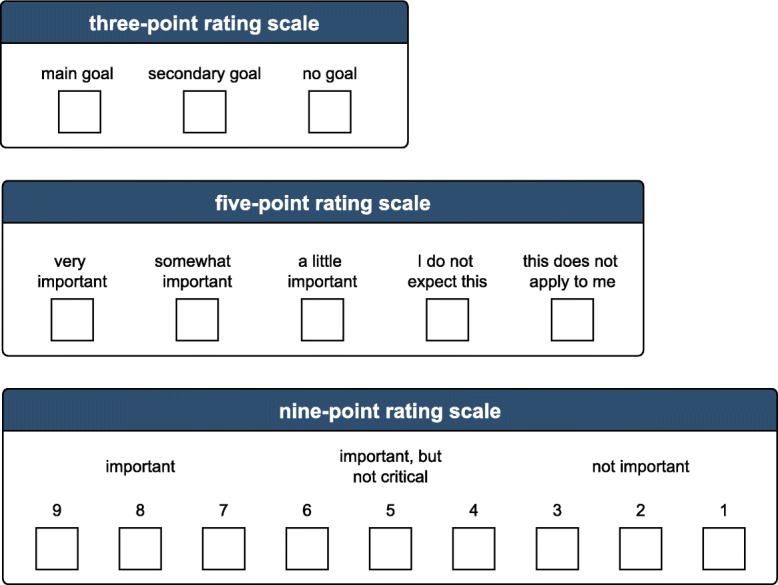
Rating scales

In the first round of the survey, 100 patients participated in the study. All of these patients were invited to participate in the second round. A total of 87 patients completed the second questionnaire (overall response-rate: 87%, Fig. 2. Characteristics of participants are summarized in Table 1. The characteristics of the patients who dropped out did not differ in sex and age from patients included in the study.

**Table 1:** Patient characteristics

	Characteristics		
	1st round	2nd round	Drop out
Sex			
Male	31 (31%)	26 (30%)	5 (38%)
Female	69 (69%)	61 (70%)	8 (62%)
No answer	0 (0%)	0 (0%)	0 (0%)
Care of relatives			
Yes	9 (9%)	9 (10%)	0 (0%)
No	87 (87%)	74 (85%)	13 (100%)
No answer	4 (5%)	4 (5%)	0 (0%)
Employment status, current			
Employed	24 (24%)	22 (25%)	2 (15%)
Unemployed	76 (76%)	65 (75%)	11 (85%)
No answer	0 (0%)	0 (0%)	0 (0%)
Age			
Mean [sd]	68.3 [± 9.9]	68.0 [± 10]	68.3 [± 9.3]
Range	42–85	42–85	48–82

### Patient preferences

The five-point (36%) was the most preferred rating scale among patients, followed by the three-point (23%) and nine-point rating scale (16%). 24% of patients rated that none of the proposed scale was preferable.

### Correlation of rating scales

Overall correlations between pairs of rating scales across all participant ratings ranged from 0.65 to 0.69 within the first, and from 0.70 to 0.74 within the second survey. After transformation of the *five-point* and *nine-point scale,* the correlation with the *three-point scale* increased to 0.74 (five-point* scale) and 0.71 (nine-point* scale) in the second survey. Depending on the specific question, correlation between pairs of rating scales ranged from 0.15 to 0.85 for each treatment goal.

### Effects of using different rating scales on consensus

#### (A) Effect of different ratings scales on the percentage main goals

Different results on the consensus were observed within the same target population, depending on the rating scale (Table 2). For a threshold of 75% which is typically used in Delphi studies in the context of COS development [13], the proportion of treatment goals falling into the top category named “*main goal”* and hence reaching consensus differed by a factor of up to two between the three scales.

**Table 2:** Consensus across different rating scales

Treatment goal: top category	Consensus on specific treatment goals					
	1st survey			2nd survey		
	three-point scale	five-point scale*	nine-point scale*	three-point scale	five-point scale*	nine-point scale*
preventing secondary impairments	82.8	69.0	90.8	85.1	70.1	89.7
duration of hospitalization	52.9	36.8	64.4	48.3	39.1	62.1
stability	94.3	79.3	97.7	96.6	81.6	96.6
pain	89.7	79.3	95.4	95.4	79.3	94.3
implant survival	79.3	75.9	89.7	86.2	73.6	83.9
quality of life	87.4	73.6	97.7	89.7	74.7	90.8
range of motion (ROM)	92.0	80.5	96.6	93.1	79.3	96.6
activity of daily life	86.2	78.2	94.3	89.7	77.0	88.5
malalignment	66.7	47.1	72.4	63.2	46.0	72.4
strength	79.3	60.9	93.1	80.5	65.5	92.0
employability	54.0	35.6	64.4	56.3	47.1	65.5
walking stairs	86.2	74.7	92.0	89.7	77.0	89.7
walking distance	93.1	75.9	96.6	92.0	75.9	94.3
no side effects	78.2	67.8	83.9	74.7	71.3	82.8
participation in social life	82.8	65.5	89.7	81.6	60.9	87.4
sex life	20.7	17.2	31.0	19.5	20.7	29.9
physical function	83.9	64.4	90.8	85.1	70.1	93.1
physical activity	71.3	59.8	88.5	70.1	70.1	80.5
global health status	90.8	73.6	94.3	89.7	79.3	92.0
Consensus threshold	Number of treatment goals that reached the threshold					
	1st survey			2nd survey		
	three-point scale (%)	five-point scale* (%)	nine-point scale* (%)	three-point scale (%)	five-point scale* (%)	nine-point scale* (%)
> 60%	16 (84.2)	14 (73.7)	18 (94.7)	16 (84.2)	15 (78.9)	18 (94.7)
> 70%	15 (78.9)	9 (47.4)	16 (84.2)	15 (78.9)	13 (68.4)	16 (84.2)
> 75%	14 (73.7)	6 (31.6)	15 (78.9)	13 (68.4)	7 (36.8)	15 (78.9)
> 80%	11 (57.9)	1 (5.3)	15 (78.9)	13 (68.4)	1 (5.3)	15 (78.9)
> 90%	4 (21.0)	0 (0)	11 (57.9)	4 (21.0)	0 (0)	8 (42.1)

The first part of the table shows the percentage of ratings as “main goal” of the 87 participants per survey across each treatment goal provided. The second part of the table shows the number and percentage of treatment goals that reached a certain level of consensus. In case of the three-point scale, the “main goal” is the top category. Five-point*/nine-point* scales: the top category of the five-point* scale, is “very important”, and for the nine-point* scale categories “9”, “8” and “7”

#### (B) Effect of different rating scales on percentage consensus using different thresholds

The *five-point scale* achieved the lowest and the *nine-point scale* the highest proportion of treatment goals that exceeded the different consensus thresholds for “*main goals”* (Table 2). This difference between the scales has increased with rising thresholds. At a threshold value set to 90% in the first survey, no consensus could be reached for the 19 goals using the *five-point scale*, whereas consensus was reached for four goals using the *three-point scale* and for 11 goals using the *nine-point scale*.

### Test-retest reliability of different rating scales

From the first survey to the second survey, 12% of all participants’ ratings changed on the *three-point*, 25% on the *five-point* and 32% on the *nine-point scale* (Table 3). The sensitivity analysis shows that after transformation, 9% of participants’ ratings were changed in the second survey on the nine-point* scale. With the exception of the nine-point* scale, participants rated the treatment goals in the second survey round with significantly higher expectations in comparison to the first survey.

**Table 3:** Inter-individual comparison of rating scales

Statistic	three-point scale	five-point scale	nine-point scale	Sensitivity analysis	
				five-point scale^a^	nine-point scale^a^
Overall^b^					
Changes in 2nd survey (in %)	12.48	24.73	32.26	20.96	8.57
Class imbalance^a^ 1st survey (in %)	79.16	64.93	63.62	64.93	88.25
Test-retest agreement (in %)	87.52	75.27	67.74	79.04	91.43
Weighted kappa [95% CI]	0.63 [0.62; 0.64]	0.47 [0.07; 0.86]	0.78 [0.78; 0.78]	0.54 [0.50; 0.58]	0.58 [0.55; 0.62]
Mean [range] over the 19 proposed treatment goals					
Changes in 2nd survey (in %)	12.60 [2.41; 25.61]	24.75 [16.05; 38.82]	32.43 [17.07; 55.13]	20.96 [16.05; 28.24]	8.69 [0.00; 24.00]
Class imbalance^c^ 1st survey (in %)	0.80 [49.38; 95.35]	66.05 [35.71; 83.13]	63.46 [21.25; 81.18]	68.65 [45.78; 83.13]	88.19 [37.50; 100.00]
Test-retest agreement (in %)	87.40 [74.39; 97.59]	75.25 [61.18; 83.95]	67.57 [44.87; 82.93]	79.04 [71.76; 83.95]	91.31 [76.00; 100.00]
Weighted kappa	0.55 [0.18; 0.87]	0.44 [0.29; 0.62]	0.61 [0.17; 0.81]	0.49 [0.35; 0.67]	0.40 [0.00; 0.80]

^a^Rating scale mapped onto three categories

^b^Overall refers total ratings of all participants of all treatment goals, e.g., the number of participants times 19 goals times ratings of the respective scale five-point/nine-point scale

^c^Class imbalance is highlighted by the percentage of the most frequently used rating category (e.g. in the first survey, the rating categories main goal/secondary goal/no goal scored 79%/11%/10% across all participants’ ratings of all goals, hence, the imbalance is 79%)

**Fig. 3:**
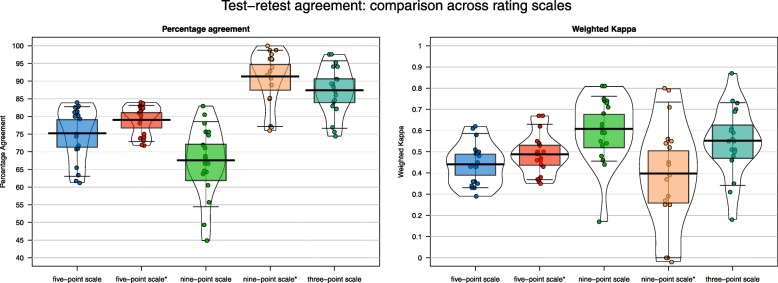
Test-retest agreement: comparison across rating scales: Pirateplot of the percentage agreement and the weighted kappa distributions between survey 1 and survey 2. Each data point represents the value for a single treatment goal. The beans represents the smoothed density, the boxes the 95% confidence interval with the middle line as the average. Five-point/nine-point scale*: rating scale mapped onto three categories: - “v*ery important*” (*five-point scale*) and “9, 8, 7” (*nine-point scale*) as “*main goal”* - “a *little important*”, “*somewhat important*” (*five-point scale*) and “5, 6, 7” (*nine-point scale*) as “*secondary goal*” -“*I do not expect this*” and “*this does not apply to me*” (*five-point scale)* and “*3, 2, 1*” (*nine-point scale*) as “*no goal*”

The prevalence of the most frequently rated category differed between rating scales. The rating category with the highest importance was selected in 65–80% of patient ratings, with a high heterogeneity across the 19 individual treatment goals (Fig. 3).

The overall weighted kappa across all participants’ ratings ranged from 0.63 to 0.78 between the proposed rating scales (Table 3). The *nine-point scale* reached the highest weighted kappa value. The sensitivity analysis shows an increase in the percentage of agreement which comes from the reduction of categories of the five-point and nine-point scale. In contrast, the weighted kappa is not as strongly affected by the transformation due to the class imbalance. The transformed *nine-point scale* shows slightly higher percentage test-retest agreement in relation to the *three-point scale*. None of the proposed scales shows a general superiority according to absolute and relative reliability measures.

## Discussion

### Main findings

#### Effects of using different rating scales on consensus

This study shows that, within the same population, the use of different rating scales (*three-point*, *five-point* and *nine-point rating scale*) lead to different consensus, despite the moderate to high correlation between the rating scales. The difference in the behavior indicates that the result of a process for finding consensus is highly affected by both, the criteria to reach consensus and to the rating scale. The effects of different thresholds on the final consensus also differs between scales. Between the two extreme scenarios (threshold values of 60 and 90%) in the five-point scale, 15 (60%) to 0 (90%) treatment goals reached the consensus (Table 2). In contrast, the *nine-point scale* in the first survey leads to a range of 11 to 18 treatment goals reaching consensus within these scenarios.

In addition to the use of different threshold values, the aggregation method of the rating scales has considerable influence on the resulting consensus. In light of this, we share the call of Grant, et al. [15] for the essential need of pre-registration and pre-defined analysis plans for Delphi studies. There is a substantial backlog in this area of medical research, especially with regard to the large impact of consensus processes on healthcare topics such as guideline development. Furthermore, we would like to highlight the importance of careful instruction of participants, feedback loops including argument lists, and effort to ensure participants’ understanding of the consensus criteria.

#### Test-retest reliability of different rating scales

We additionally compared the test-retest reliability of three rating scales with different metrics and different anchors. As a result, none of the three rating scales can be selected based on the investigated psychometric test properties alone, as none of the rating scales is substantially superior to the others with respect to the reliability. Between single questions, we observed a wide range of reliability values regarding the importance of the single treatment goals, which indicates uncertainty among patients evaluating the importance of expected treatment goals. This result highlights the need for feedback loops and providing argument lists in Delphi studies.

#### Implication for the use case consensus of treatment goals on TKA

It is impossible to recommend one of the investigated rating scale solely due to the test-retest reliability or the stability of consensus results choosing different thresholds without considering the clinical context. The decision which rating scale should be used, needs to be critically discussed in accordance with the specific purpose, the expected outcome of the consensus procedures and the measurement properties of the rating scales.

In the context of treatment goals for TKA, it is clinically necessary to distinguish between a treatment goal that must be achieved with a direct clinical implication, in comparison to questioning the relative importance of treatment goals such as the *five-point* and *nine-point scale*. Therefore, in our setting to develop a set of global treatment goals, we preferred the *three-point scale* because further translations/transformations of importance into a clinical context of “*main goals”* were not required. However, there is no gold standard to evaluate the accuracy of this scale. We conclude that in a consensus-orientated Delphi study, the used rating scale should reflect the context-based research question.

### Methodological considerations

#### Effects of using different rating scales on consensus

In many consensus-orientated Delphi studies, dichotomous decisions (agreement/rejection; inclusion/exclusion) were generated from a *nine-point scale*. This raises the question why the ordinal scale is necessary in consensus-orientated Delphi studies. It may be useful to get a first impression of a group opinion with a wide range rating scale (e.g. *nine-point scale*), but finally a consensus-oriented Delphi process always ends with a dichotomous result (e.g. main goal, core outcome, recommendation). This topic needs to be investigated in other areas, regardless of the topic of treatment goals. Finally, it remains unresolved whether it is better to define a scale-cutoff and then generate a dichotomous result or whether that result should be queried in a context-based dichotomous manner, e.g. whether one should formulate all questions in a yes/no manner.

#### Test-retest reliability of different rating scales

Based on the results from this study, large differences between ratings on individual treatment goals exists in comparison of test and retest. Thus, the validity of consensus process should be questioned if reliability is insufficient, since an instrument that is not reliable, cannot be valid by definition at all [41]. Since there is no equivalent to a Bland and Altman plot [42] for ordinal scales of different characteristics, we had to use absolute and relative reliability statistics for the comparison of different scales. Kappa coefficients should ideally reach values over 0.70 [43]. However, the prevalence effects (preferred selection of one category resulting in a prevalence that differs from uniform distribution) reduce the value of the kappa statistics [44–46] and increases the divergence between absolute and relative reliability. Patients frequently rated the proposed treatment goals with high importance. This results in a high number of ratings in one category (class imbalance) which influences the relative reliability measures. Despite this, there are very similar problems of class imbalance in other areas where Delphi studies are common (e.g. COS development). Therefore, both statistics (percentage agreement, weighted kappa) were interpreted jointly in this study.

### Limitations

In this study, we investigated the influence of different rating scales on the outcome of a Delphi process in the field of treatment goals in elective orthopedic surgery with untrained German patients. The results might be different with trained participants or in other countries or cultures.

Despite our efforts to reduce bias by randomizing the order of rating scales and the questions within a question block, we cannot estimate to which extent patients have been influenced by the order of questions and scales. Due to feasibility aspects, study participants saw and rated the treatment goals on all three scales simultaneously, which might have introduced bias.

To allow fair comparison of the reliability between scales with different numbers of categories we mapped the five-point and nine-point scales to three-point scale. The aim of the sensitivity analysis was to enable comparison between scales. However, one has to keep in mind that the results for the respective categories depend on the transformation. Hence, the strategy should be used to identify trends in reliability measures rather than direct comparison of categories.

Patient treatment goals may have changed between the two survey rounds, although this is unlikely because the time between both rounds was restricted to 14 days or less. It can also not be ruled out that patients changed their decision in the meantime rather than responding to the second questionnaire with their original goals in mind, resulting in a reduced test-retest agreement with previous ratings. Furthermore, some of the people may have completed the second assessment with a minimal of 2 days after the first one. Hence, there is a chance of a memory effect, which, in our case would lead to an underestimation of response variability.

In this study, no feedback regarding previous ratings was given during the second survey, which might have influenced the proportion of treatment goals reaching consensus, and differs from recommendations for Delphi consensus processes were feedback on own and group ratings is recommended [13]. In addition, no summary of the arguments for or against a treatment goal was collected in the surveys. However, in a Delphi consensus process with feedback this is an essential part and could also have a significant impact on the validity.

### Implication for further research

The importance of accessing patient expectations in the context of shared-decision making and evidence-based medicine [47] and formulation of the corresponding treatment goals is constantly growing in modern health care. Carefully designed Delphi studies including patient expectations should increasingly be used to reach consensus in multi-perspective studies in the context of guideline and COS development or similar processes, to address patient perspectives in healthcare research. Unfortunately, to our knowledge, the involvement of patient expectations is rarely used in medical research.

It is essential to measure consensus with appropriate rating scales and to expand research in this area accordingly. Further research investigating the appropriateness of different rating scales should simultaneously examine different methods for the definition of the final consensus. There is no global approach to the criteria that define consensus [9]. Due to the design of the study to verify test-retest reliability, patients did not receive anonymous group feedback. Therefore, further research is needed to investigate rating scale properties and the influence of rating scales regarding the resulting proportion of items reaching consensus in Delphi studies with the iterative feedback loop. The investigation of appropriate rating scales should be extended to other relevant areas, such as COS development.

Furthermore, research is needed to examine whether an online survey alone is sufficient to reach consensus. In an online survey the possibilities of an (open) discussion between participants are usually limited and there is no further training to understand threshold values or the chosen rating scale. The presentation of argumentation lists for or against statements can help to improve the validity of the consensus process in Delphi studies. In contrast, in a split approach involves prioritization within an online survey and afterwards the final consensus will be reached via face-to-face meetings with the possibilities for discussion and training [13]. Comparative research for these two approaches is essential to assess the validity of final consensus.

## Conclusion

In addition to already known factors influencing the results of Delphi processes, this study provides evidence that a consensus also depends on the format of rating scale and consensus threshold. Investigators and participants of consensus studies need to be aware that the nature of the scale has a high impact on the results of a consensus study. It is yet unclear to what extent these findings are generalizable to Delphi studies conducted among experts or Delphi studies targeting objectives other than treatment goals.

The test-retest reliability of the three rating scales investigated differs substantially between individual treatment goals. Large variation in reliability implies that there could be a substantial proportion of treatment goals with low reliability and hence low validity. Thus, this variation introduces a potential source of bias in consensus studies that researchers should be aware of. However, we found no clear evidence of the superiority of one scale based on reliability.

In summary, the selection of rating scales and corresponding consensus thresholds should base on the specific context, expected outcome and scale property aspects. To capture patients’ treatment goals for TKA, the *three-point scale* (“*main goal*”, “*secondary goal*” and “*no goal*”) was preferred, since further reclassification or translation into the clinical context with clinical implication was not required.

## Supplementary information

**Additional file 1:** Treatment goal questionnaire (The questionnaire specifically developed for this study).

## Data Availability

The datasets used and/or analyzed during the current study are available from the corresponding author on reasonable request.

## Abbreviations

COS: Core outcome set
EKIT: Evidence and consensus based indication for total knee arthroplasty
k: Kappa coefficient
OA: Osteoarthritis
ROM: Range of motion
TKA: Total knee arthroplasty
