# Supplementary material for: Comparison of different rating scales for the use in Delphi studies: different scales lead to different consensus and show different test-retest reliability
Source: BMC Med Res Methodol. 2020 Feb 10;20:28. doi: 10.1186/s12874-020-0912-8 (PMC7011537; doi:10.1186/s12874-020-0912-8)
Supplement: Supplementary file 1 — Additional file 1: Treatment goal questionnaire (The questionnaire specifically developed for this study). [file 12874_2020_912_MOESM1_ESM.pdf]

Translated version; original language: German

EKIT-Knie

Treatment goal questionnaire

B: Survey on treatment goals (1)

Please read the following questions carefully and, if possible, answer all questions by ticking the appropriate answer fields. In case of doubt, please tick the most appropriate answer. Please note that you may only place **ONE cross** per question.

Please determine whether the goals listed below are a "main goal", a "secondary goal" or possibly "no goal" for you, considering treatment of your knee osteoarthritis.

A **"main goal"** MUST be achieved to consider the operation as successful for you.

A **"secondary goal"** is DESIRABLE, but needs not necessarily to be achieved to consider the operation as successful for you.

"No goal" is not relevant or not important to you.

**Are the following terms a goal for you if you are thinking of treating your osteoarthritis of the knee joint with an artificial joint?** ***(Please note that you may only place ONE cross for each of the objectives listed below!)***		**Main goal**	**Secondary goal**	**No goal**
**B1:**	Improving **employability**	◻	◻	◻
**B2:**	**High quality of life** (physical, mental and social well-being)	◻	◻	◻
**B3:**	**Improving the walking distance** (being able to walk a certain distance)	◻	◻	◻
**B4:**	**Pain relief**	◻	◻	◻
**B5:**	**Strength of the leg muscles**	◻	◻	◻
**B6:**	**Stability of the knee**	◻	◻	◻
**B7:**	Improving **physical function** (sitting down, kneeling)	◻	◻	◻
**B8:**	**Implant survival** (duration until a replacement operation)	◻	◻	◻
**B9:**	Normal leg **alignment** (no X-leg or O-leg)	◻	◻	◻
**B10:**	Improving **physical activity** (gardening, cycling)	◻	◻	◻
**B11:**	No **side effects**	◻	◻	◻
**B12:**	Ability to carry out **activity of daily life** (e.g. personal hygiene, use of public transport)	◻	◻	◻
**B13:**	Improving/maintaining your **sexual life**	◻	◻	◻
**B14:**	Good knee **range of motion**	◻	◻	◻

Translated version; original language: German

Are the following terms a goal for you if you are thinking of treating your osteoarthritis of the knee joint with an artificial joint? *(Please note that you may only place ONE cross for each of the objectives listed below!)*		Main goal	Secondary goal	No goal
B15:	Improving/maintaining the **general health status** (complete physical, mental and social well-being and not merely the absence of disease or disability)	□	□	□
B16:	Short **duration of hospitalization**	□	□	□
B17:	**Participation in social life** (joint activities with family and friends)	□	□	□
B18:	Improving **walking stairs**	□	□	□
B19:	Reduction/prevention of **secondary impairments** of osteoarthritis (overloading the other leg, back pain)	□	□	□

C: Survey on treatment goals (2)

Please read the following questions carefully and, if possible, answer all questions by ticking the appropriate answer fields. In case of doubt, please tick the most appropriate answer. Please note that you may only place **ONE cross** per question.

Please determine whether the goals listed below are a "very important", "somewhat important", "a little important", "I do not expect this", "I do not expect this" for you, considering treatment of your knee osteoarthritis.

If none of the answer options ("very important", "somewhat important", "a little important", "I do not expect this") is possible for you, or you are unable to judge this, please place your cross at "this does not apply to me".

Are the following terms a goal for you if you are thinking of treating your osteoarthritis of the knee joint with an artificial joint? *(Please note that you may only place ONE cross for each of the objectives listed below!)*		Very important	Somewhat important	A little important	I do not expect	This does not apply to me
C1:	Good knee **range of motion**	□	□	□	□	□
C2:	Ability to carry out **activity of daily life** (e.g. personal hygiene, use of public transport)	□	□	□	□	□
C3:	**Pain relief**	□	□	□	□	□
C4:	Normal leg **alignment** (no X-leg or O-leg)	□	□	□	□	□
C5:	Short **duration of hospitalization**	□	□	□	□	□
C6:	**Stability of the knee**	□	□	□	□	□
C7:	Reduction/prevention of **secondary impairments** of osteoarthritis (overloading the other leg, back pain)	□	□	□	□	□
C8:	Improving/maintaining your **sexual life**	□	□	□	□	□
C9:	Improving **physical function** (sitting down, kneeling)	□	□	□	□	□

Translated version; original language: German

**Are the following terms a goal for you if you are thinking of treating your osteoarthritis of the knee joint with an artificial joint?**<br><br>***(Please note that you may only place ONE cross for each of the objectives listed below!)***		**Very important**	**Somewhat important**	**A little important**	**I do not expect**	**This does not apply to me**
**C10:**	Improving **physical activity** (gardening, cycling)	□	□	□	□	□
**C11:**	**Implant survival** (duration until a replacement operation)	□	□	□	□	□
**C12:**	Improving **walking stairs**	□	□	□	□	□
**C13:**	**Participation in social life** (joint activities with family and friends)	□	□	□	□	□
**C14:**	**Strength of the leg muscles**	□	□	□	□	□
**C15:**	**High quality of life** (physical, mental and social well-being)	□	□	□	□	□
**C16:**	**Improving the walking distance** (being able to walk a certain distance)	□	□	□	□	□
**C17:**	Improving **employability**	□	□	□	□	□
**C18:**	Improving/maintaining the **general health status** (complete physical, mental and social well-being and not merely the absence of disease or disability)	□	□	□	□	□
**C19:**	No **side effects**	□	□	□	□	□

D: Befragung zu Therapiezielen (3)

Please read the following questions carefully and, if possible, answer all questions by ticking the appropriate answer fields. In case of doubt, please tick the most appropriate answer. Please note that you may only place **ONE cross** per question.

Please determine how important you consider the following goals to be when you think of the treatment of your knee osteoarthritis. Please rate the importance of the mentioned goals using the scale from "9" to "1" in descending order, where "9" is most important and "1" is not important at all.

**Are the following terms a goal for you if you are thinking of treating your osteoarthritis of the knee joint with an artificial joint?**<br>***(Please note that you may only place ONE cross for each of the objectives listed below!)***		**Important**			**Important, but not critical**			**Not important**		
		**9**	**8**	**7**	**6**	**5**	**4**	**3**	**2**	**1**
**D1:**	Reduction/prevention of **secondary impairments** of osteoarthritis (overloading the other leg, back pain)	□	□	□	□	□	□	□	□	□
**D2:**	**Participation in social life** (joint activities with family and friends)	□	□	□	□	□	□	□	□	□
**D3:**	Normal leg **alignment** (no X-leg or O-leg)	□	□	□	□	□	□	□	□	□
**D4:**	No **side effects**	□	□	□	□	□	□	□	□	□

Translated version; original language: German

**Are the following terms a goal for you if you are thinking of treating your osteoarthritis of the knee joint with an artificial joint?** ***(Please note that you may only place ONE cross for each of the objectives listed below!)***		**Important**			**Important, but not critical**			**Not important**		
		**9**	**8**	**7**	**6**	**5**	**4**	**3**	**2**	**1**
**D5:**	Ability to carry out **activity of daily life** (e.g. personal hygiene, use of public transport)	□	□	□	□	□	□	□	□	□
**D6:**	Improving/maintaining the **general health status** (complete physical, mental and social well-being and not merely the absence of disease or disability)	□	□	□	□	□	□	□	□	□
**D7:**	Improving **physical activity** (gardening, cycling)	□	□	□	□	□	□	□	□	□
**D8:**	Improving/maintaining your **sexual life**	□	□	□	□	□	□	□	□	□
**D9:**	**Pain relief**	□	□	□	□	□	□	□	□	□
**D10:**	**Stability of the knee**	□	□	□	□	□	□	□	□	□
**D11:**	Good knee **range of motion**	□	□	□	□	□	□	□	□	□
**D12:**	**Improving the walking distance** (being able to walk a certain distance)	□	□	□	□	□	□	□	□	□
**D13:**	Improving **employability**	□	□	□	□	□	□	□	□	□
**D14:**	Short **duration of hospitalization**	□	□	□	□	□	□	□	□	□
**D15:**	Improving **walking stairs**	□	□	□	□	□	□	□	□	□
**D16:**	**Strength of the leg muscles**	□	□	□	□	□	□	□	□	□
**D17:**	Improving **physical function** (sitting down, kneeling)	□	□	□	□	□	□	□	□	□
**D18:**	**Implant survival** (duration until a replacement operation)	□	□	□	□	□	□	□	□	□
**D19:**	**High quality of life** (physical, mental and social well-being)	□	□	□	□	□	□	□	□	□

For which of the three types of questioning (B, C or D) was it easiest for you to decide on an answer?

(Please tick only ONE of the following answer options)

□ Survey B (3 possible answers)
□ Survey C (5 possible answers)
□ Survey D (9 possible answers)
□ With none (all equally good/equally bad)
